# *Tisochrysis lutea* Fucoxanthin Suppresses NF-κB, JNK, and p38-Associated MMP Expression in Arthritis Pathogenesis via Antioxidant Activity

**DOI:** 10.3390/antiox13080941

**Published:** 2024-08-02

**Authors:** Hyemi Lee, Hahyeong Jang, Dahyoon Heo, Jae-In Eom, Cheol-Ho Han, Se-Min Kim, Yoo-Seob Shin, Cheol-Ho Pan, Siyoung Yang

**Affiliations:** 1 Department of Biological Sciences, Sungkyunkwan University, Suwon 16419, Republic of Korea; hyemi0320@skku.edu (H.L.); jhh7071@g.skku.edu (H.J.); hdy0523@g.skku.edu (D.H.); 2 Microalgae Ask Us Co., Ltd., Gangneung 25441, Republic of Korea; umjaein@maus2020.com (J.-I.E.); hancheolho@maus2020.com (C.-H.H.); kimsemin@maus2020.com (S.-M.K.); shinyooseob@maus2020.com (Y.-S.S.)

**Keywords:** *Tisochrysis lutea*, fucoxanthin, antioxidant, catabolic factors, osteoarthritis

## Abstract

*Tisochrysis lutea* is a highly nutritious marine microalga that has various applications in aquaculture and biotechnology. However, the effects of *T. lutea* extract (TLE) on osteoarthritis (OA) pathogenesis remain unexplored. In this study, we aimed to determine the effects of TLE on OA development. We found that TLE inhibits the expression of matrix metalloproteinases (MMPs) and reactive oxygen species (ROS) activity in an OA mouse model generated by the destabilization of the medial meniscus (DMM) surgery. In vivo assays of the OA model mice demonstrated that TLE has a protective effect against cartilage destruction by inhibiting MMP3 and MMP13 expression. To enable the medical use of TLE, the components of TLE were characterized using high-performance liquid chromatography (HPLC) analysis. Interestingly, we found that Fucoxanthin accounts for 41.2% of TLE and showed anti-catabolic and antioxidant effects under IL-1β-treated in vitro conditions. RNA sequencing analysis showed that fucoxanthin decreased p38, NF-κB, and JNK signaling pathway gene expression, all of which are activated by IL-1β. Furthermore, in vivo analysis showed that fucoxanthin inhibited the IL-1β-stimulated phosphorylation of p65, JNK, and p38. These results highlight new possibilities for the use of TLE as a source of fucoxanthin, an antioxidant, for OA treatment.

## 1. Introduction

Osteoarthritis (OA) is a major type of arthritis marked by the progressive breakdown of joint cartilage and the bone beneath it, leading to discomfort, stiffness, and limited movement [1,2]. The development of OA involves multiple factors, including mechanical, biological, biochemical, and genetic influences [3]. The development and progression of OA at the molecular level are influenced by the intricate interactions between pro-inflammatory cytokines, matrix-degrading enzymes, and cellular oxidative stress [4,5,6,7]. The disruption of the equilibrium between anabolic and catabolic activities is an important aspect of the pathogenesis of OA. During OA pathogenesis, cells undergo phenotypic alterations that enhance the synthesis of matrix metalloproteinases (MMPs), a group of zinc-dependent enzymes, resulting in the breakdown of the extracellular matrix (ECM) by the formation of reactive oxygen species (ROS) [3,4,8]. In addition, the inflammatory conditions within the joint are intensified by the secretion of cytokines like interleukin-1β (IL-1β) and tumor necrosis factor-α (TNF-α), which also promote destructive processes and hinder the body’s ability to repair itself [9,10].

The production of MMPs in osteoarthritis is significantly regulated by the nuclear factor kappa-light-chain-enhancer of activated B cells (NF-κB) and mitogen-activated protein kinase (MAPK) pathways [3,8,9,11,12]. The activation of these pathways is triggered by pro-inflammatory cytokines and mechanical stress, resulting in the transcription of genes responsible for the breakdown of the ECM and oxidative stress [4,8,11]. Canonical IL-1β-mediated activation of transcription factor NF-κB through TRAF6-mediated p65 and IκBα phosphorylation drives MMP3 and MMP13 expression. By exploiting a zinc catalyst, members of the MMP family remodel tissue through proteolysis [8,11,13,14,15]. Similarly, the phosphorylation of MAPK and JNK pathways by pro-inflammatory cytokines (such as IL-1β) drives the expression of MMPs, which are, in turn, activated by ROS [12,14]. Specifically, the activation of the JNK and p38 pathways results in the upregulation of MMP expression [14,16,17]. Thus, improving our understanding of these pathways may provide valuable insights into possible treatment targets for controlling OA by reducing MMP activation [8,18].

*Tisochrysis lutea* (*T. lutea*), a haptophyte algae important in shellfish aquaculture, generates compounds such as fucoxanthin (a xanthophyllic carotenoid pigment with antioxidant activity) and docosahexaenoic acid that are beneficial to human health [19]. Previous studies have demonstrated its potential for the treatment and prevention of numerous diseases, making it an important focus for further investigations and drug development [19,20,21,22]. *T. lutea* aids in the neutralization of free radicals, which may decrease the risk of chronic illnesses, such as cancer and cardiovascular ailments [19]. In addition, *T. lutea* extract (TLE) has anti-inflammatory, anticancer, and neuroprotective activities against various diseases [23,24,25]. In particular, this substance is recognized for its carotenoid content, including fucoxanthin, which exhibits strong anticancer effects. Xanthin pigments have been reported to possess antioxidant, anti-inflammatory, anti-obesogenic, and anti-carcinogenic effects in vitro and in vivo [20,24,26,27]. Thus, fucoxanthin from TLE may exhibit significant anti-OA potential through the amelioration of MMP-activating ROS.

In this study, we determined the therapeutic effect of TLE and its component, fucoxanthin, on OA development. Our findings indicate that both TLE and its component function as novel therapeutics to mitigate the progression of OA by suppressing the expression of catabolic factors through the regulation of genes associated with the NF-κB, JNK, and p38 pathways.

## 2. Materials and Methods

### 2.1. Sample Treatment and Reagents

TLE and fucoxanthin were provided by Microalgae Ask Us Co., Ltd. (Gangneung, Republic of Korea). To obtain the total extract of *T. lutea*, 100 g of freeze-dried *T. lutea* was mixed with ethyl acetate at a ratio of 1:10 (*w*/*v*). Then, the mixture was extracted at 25 °C for 1 h. The extract was subsequently filtered using a vacuum rotary evaporator under low pressure and was concentrated. The fractionation process was repeated thrice using a solvent mixture consisting of 70% ethanol and n-hexane in a 1:1 (*v*/*v*) ratio. Following fractionation, the ethanol layer, which accounted for 70% of the recovered solution, was concentrated and freeze-dried to obtain a fraction of about 2 g. This resulting complete TLE was subsequently evaluated using high-performance liquid chromatography (HPLC). To prepare fucoxanthin, 100 g of silica gel (230–400 mesh, Merck, Palo Alto, CA, USA) was suspended in a solution of n-hexane/acetone (7:3, *v*/*v*). The suspension was then packed into a glass column (DURAN, Mainz, Germany). The entire extract of *T. lutea* (approximately 2 g) was added to the column and eluted in the dark. The crimson layer was gathered in test tubes and condensed to obtain a 95% fucoxanthin sample, which was examined using HPLC. The samples were first dissolved in dimethyl sulfoxide (DMSO) and subsequently heated at 55 °C for 15 min. The recombinant human IL-1β protein (Z02922-10; GenScript, Piscataway, NJ, USA) was then prepared in phosphate-buffered saline (PBS) as a vehicle. The samples were treated with the appropriate dose (1 ng/mL) of IL-1β in IL-1β-treated chondrocytes 12 h before cell harvesting, as specified in each figure legend. All solvents used to extract, fractionate, and purify fucoxanthin from *T. lutea* were ACS grade (≥95%).

### 2.2. Analysis of the EtOAc Fraction from T. lutea Using HPLC

An Agilent 1260 HPLC system (Agilent Technologies, Santa Clara, CA, USA) was utilized for HPLC analysis. A CAPCELL PAK C18 MG II column (5 μm particle size, 250 mm × 4.6 mm) was employed. The mobile phase, comprising analytical-grade acetonitrile (A) and water (B), flowed at 1 mL/min. The phase ratio was adjusted from 90:10 to 100:0 (A to B) over 8 min, held at 100:0 for 3 min, and then reduced to 80:20 over 5 min. Chromatograms were recorded at a 450 nm wavelength. Fucoxanthin levels were determined by measuring peak areas in the chromatograms against a standard curve (1, 5, 10, 50, 100, and 200 μg/mL) using a fucoxanthin standard from Sigma Aldrich (St. Louis, MO, USA).

### 2.3. Primary Culture of Mouse Chondrocytes and Experimental Animals

Male C57BL/6J mice, 10 weeks old, were sourced from DBL (Chungcheongbuk-do, Eumseong, Republic of Korea). Each in vitro experiment was conducted a minimum of four times. In vivo experiments involving animals received approval from the Sungkyunkwan University Animal Care and Use Committee under the protocol code SKKUIACUC2023-07-21-1, adhering to the 8th edition of the NIH’s Guide for the Care and Use of Laboratory Animals (protocol code 2016-0041). Additionally, both C57BL/6 and 5-day-old Institute of Cancer Research (ICR) mice were obtained from DBL Co., Ltd. (Chungbuk, Republic of Korea). These mice, weighing 18–20 g, were kept in a controlled environment at 23 °C with a 12/12 h light–dark cycle, with consistent access to water and food. They were used to create a destabilized medial meniscus (DMM) mouse model. Articular chondrocytes were isolated from the femoral condyles and tibial plateaus of 5-day-old ICR mice. The chondrocytes were extracted using Type II collagenase and cultured in DMEM enriched with 1% penicillin/streptomycin and 10% fetal bovine serum (FBS; Capricorn, Ebsdorfergrund, Germany), using cells from passage 0 for all experiments [17].

### 2.4. Lactate Dehydrogenase (LDH) Assay

A lactate dehydrogenase (LDH) assay was performed to assess the cytotoxic effects of TLE and fucoxanthin on chondrocytes. Roughly 24 h before cell collection, the culture medium was switched to one containing 0% FBS, and the cells were exposed to TLE or fucoxanthin at 50, 100, and 200 μg/mL. The LDH activity in the chondrocyte supernatant was measured. Cytotoxicity levels were calibrated against control samples showing 0% cytotoxicity and those treated with Triton X-100 showing 100% cytotoxicity. Cell survival was calculated according to the manufacturer’s protocols. Absorbance readings were taken at 490 nm with a SYNERGY H1 microplate reader (BioTek, Winooski, VT, USA).

### 2.5. Reverse Transcription–Polymerase Chain Reaction (RT-PCR) and Quantitative RT-PCR (qRT-PCR)

TRIzol reagent (Molecular Research Center Inc., Cincinnati, OH, USA) was used to extract total RNA from mouse chondrocytes according to the manufacturer’s instructions. The total RNA was converted into complementary DNA (cDNA) using an Im-Prom-IITM Reverse Transcriptase kit (A3803; Promega, Madison, WI, USA) and oligo-dT primers. The primer and PCR conditions used for each gene were as follows: for mouse MMP3 (NM_010809), the primers used were 5′-CTGTGTGTGGTTGTGTGCTCATCCTAC-3′ and 5′-GGCAAATCCGGTGTATAATTCACAATC-3′, with an annealing temperature of 58 °C and 21 cycles; for mouse MMP13 (NM_008607), the primers used were 5′-TGATGGACCTTCTGGTCTTCTGGC-3′ and 5′-CATCCACATGGTTGGGAAGTTCTG-3′, with an annealing temperature of 58 °C and 21 cycles; and for mouse glyceraldehyde 3-phosphate dehydrogenase (GAPDH) (NM_001289726), the primers used were 5′-TCACTGCCACCCAGAAGAC-3′ and 5′-TGTAGGCCATGAGGTCCAC-3′, with an annealing temperature of 58 °C and 21 cycles. To measure gene transcription levels, we conducted qRT-PCR using SYBR premix Ex Taq (Takara Bio, Shiga, Japan). The relative levels of gene expression were analyzed using the 2^−ΔΔCt^ method and standardized by comparison with GAPDH levels.

### 2.6. Collagenase Assay

Collagenase activity was measured from the conditioned media of the articular chondrocyte culture by utilizing an EnzCheck Gelatinase/collagenase Assay Kit (Molecular Probes, Carlsbad, CA, USA) and a SYNERGY H1 microplate reader (BioTek, Winooski, VT, USA) at 490 and 530 nm for excitation and emission, respectively. All of the processes followed the manufacturer’s protocol. 

### 2.7. Western Blot and Densitometry Analysis

Proteins were extracted from mouse chondrocytes using a lysis buffer (containing 150 mM NaCl, 50 mM Tris, 0.2% sodium dodecyl sulfate, 1% NP-40, and 5 mM NaF) supplemented with a protease inhibitor cocktail (Roche, Madison, WI, USA). MMP3 and MMP13 proteins were precipitated from the culture medium of mouse chondrocytes using trichloroacetic acid. Following size separation by acrylamide gel electrophoresis, the proteins were transferred to a PVDF membrane. The primary antibodies applied included mouse anti-ERK (sc-514302; Santa Cruz, Dallas, TX, USA), rabbit anti-MMP3 (ab52915; Abcam, Cambridge, UK), rabbit anti-MMP13 (ab51072; Abcam), and various others from Cell Signaling Technology (Danvers, MA, USA), such as mouse anti-IκB, p65, pp65, p38, pp38, JNK, pJNK, and pERK. ERK served as a housekeeping gene to normalize protein levels [17,28,29,30,31]. The band intensities were quantitatively analyzed using ImageJ software (Version 1.54j) [29].

### 2.8. In Silico Analysis; Gene Signature Enrichment Analysis (GSEA) 

Mouse chondrocytes were co-treated with IL-1β (1 ng/mL) and fucoxanthin. For in silico analyses, total RNA was isolated and analyzed using RNA sequencing. The RNA sequencing data were compared with a list of genes associated with OA, ROS, ERK, NF-κB, JNK, and p38 signaling pathways obtained from Ingenuity Pathway Analysis (IPA, http://www.ingenuity.com, accessed on 2 March 2024). The expression patterns were analyzed using GSEA (ver. 4.3.2; Broad Institute, MIT, Cambridge, MA, USA), and in silico evaluations were performed using a previously established method [32].

### 2.9. DMM-Induced OA Mouse Model and TLE Treatment

To generate the experimental OA models, 12-week-old male C57BL/6 mice underwent DMM surgery utilizing a previously published procedure [33]. For the DMM surgery, the adipose tissue was excised, and the ligament of the medial meniscus tibia was cut. This technique elevates the mechanical strain on the knee joints of mice, which can result in cartilage deterioration and the development of abnormalities resembling OA. Following DMM surgery, mice were orally administered PBS, 100 mg/kg TLE, or 100 mg/kg Boswellia once every two days for 10 weeks. Each group consisted of five animals. As a positive control, Boswellia was orally administered to mice with OA induced by DMM.

### 2.10. Histological Analysis of Cartilage Destruction and Immunohistochemistry

Cartilage samples from mice with OA were collected 10 weeks after surgery. The samples were preserved overnight in a 4% solution of paraformaldehyde and then softened in a 0.5 M EDTA solution for two weeks. Subsequently, the cartilage samples were embedded in paraffin to create paraffin blocks and sliced into sections 5 microns thick. Hematoxylin, safranin O, and quick green were used to stain the sections [17]. Histological evaluation was conducted using the OARSI (Osteoarthritis Research Society International) Grading System. A specific image of tissue stained with Safranin O was selected from a sequence of slices displaying the most advanced lesions. The OARSI scores were determined by three independent evaluators who were unaware of the group assignments. The OARSI scoring system includes seven levels from 0 to 6, where Grade 0 represents a joint with no signs of OA, and Grades 1–6 reflect various levels of cellular presence in the superficial zone and abnormalities in the cartilage matrix. Specifically, these terms refer to surface irregularities, vertical cracks, erosion, surface material removal, and changes in shape or structure [34]. Immunohistochemistry was performed to examine the cartilage protein expression of MMP3 (Abcam, Cambridge, UK), MMP13 (Abcam), and 8-OHdG (Sigma, Kawasaki-shi, Japan). The samples underwent hydration through a method akin to the one employed for safranin O staining, and then were treated with an antigen-retrieval solution, a background blocking agent, and both primary and secondary antibodies. Visualization of the samples was achieved using aminoethyl carbazole detection before their quantification was performed with ImageJ software. 

### 2.11. CellROX Staining for Live Cells

Cellular ROS was analyzed using CellROX™ Green Reagent (Invitrogen, Waltham, MA, USA). Mouse primary chondrocytes were treated with TLE and fucoxanthin along with IL-1β for 12 h. CellROX (5 μM) and DAPI (1 μg/mL) were added to the cells for 30 min before harvest. Microscopy images were acquired using an LSM980 microscope (Carl Zeiss, Baden-Württemberg, Germany). 

### 2.12. Statistical Analysis

The data are represented as the mean ± standard error. Two investigators independently prepared each histological specimen. Every experiment was replicated at least five times. The data for the two groups were analyzed using a two-tailed independent *t*-test. When more than three groups were compared, a one-way ANOVA with Bonferroni’s post hoc test was utilized, while the Mann–Whitney U test assessed non-parametric data between two groups. For multiple group comparisons, the Kruskal–Wallis test or the Friedman test was applied, followed by post hoc analysis with the Mann–Whitney U test. Results were expressed as mean ± standard deviation (SD), and a *p*-value of less than 0.05 was considered statistically significant. GraphPad Prism 9 (GraphPad, San Diego, CA, USA) was used for all statistical analyses.

## 3. Results

### 3.1. TLE Effectively Attenuates Catabolic Factor Expression in Chondrocytes

**Figure 1:**
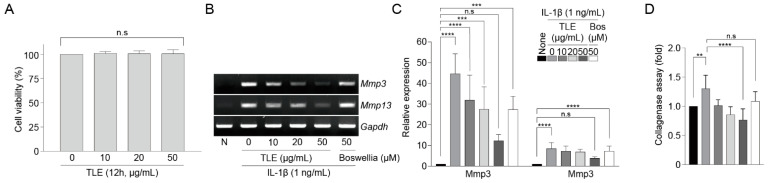
MMP3 and MMP13 expressions were downregulated by TLE in IL-1β-treated mouse articular chondrocytes. Chondrocytes were treated with TLE (10, 20, and 50 ng/mL) with or without IL-1β treatment (**A**–**D**). (**A**) Toxicity of TLE in chondrocytes. Cell viability was evaluated using a lactate dehydrogenase assay after 12 h of incubation. (**B**,**C**) The expression levels of MMP3, MMP13, and IL-6 were examined using PCR and quantified through qRT-PCR (*n* = 5). (**D**) Collagenase activity of MMPs was measured using a collagenase assay kit. The data are shown as the mean ± standard deviation (SD) and were analyzed using one-way ANOVA with Dunnett’s multiple comparisons test. (*n* = 5). ** *p* < 0.01, *** *p* < 0.001, **** *p* < 0.0001, and n.s. = not significant. TLE: *Tisochrysis lutea* extract.

To assess the cytotoxicity of TLE, mouse articular chondrocytes were exposed to various concentrations of TLE for a duration of 24 h. An LDH assay revealed that doses of up to 50 µg/mL had no influence on the viability of chondrocytes (Figure 1A), indicating that concentrations of TLE up to 50 µg/mL are safe for chondrocytes. IL-1β is a pro-inflammatory cytokine that is essential in OA development, contributing to the breakdown of cartilage by inducing the expression of MMP3 and MMP13 [8,15,16]. Therefore, we subsequently investigated whether TLE inhibits the expression of pathogenic factors under IL-1β-treated in vitro conditions. TLE suppressed the expression of MMP3 and MMP13, as shown in Figure 1B and 1C, respectively. Subsequently, we investigated whether TLE reduced MMP activity. MMP3 possesses collagenase activity and degrades type II collagen, aggrecan, and other components of the ECM. TLE significantly inhibited the IL-1β-induced increase in collagenase activity in chondrocytes (Figure 1D). Additionally, it reduced the collagenase activity of these enzymes in a dose-dependent manner (Figure 1D). Next, we explored whether these IL-1β-treated in vitro phenotypes were similar to those observed in the in vivo OA mouse experiments. 

### 3.2. The Oral Administration of TLE Inhibited Cartilage Degradation in the DMM-Induced Osteoarthritis Model

**Figure 2:**
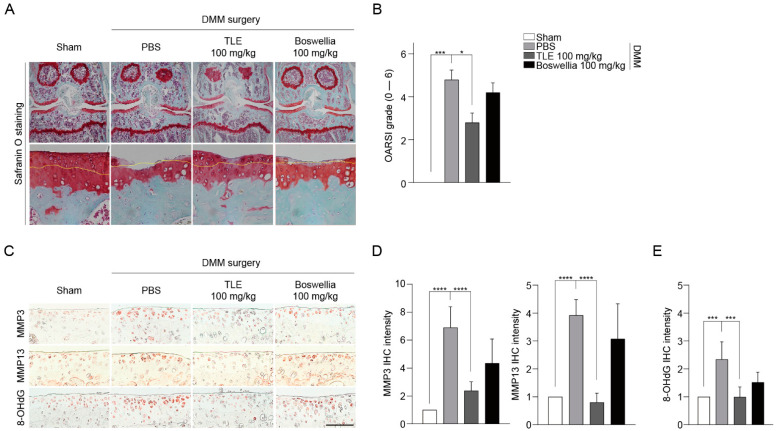
Oral administration of TLE lessened cartilage damage in mice with OA induced by DMM (*n* = 5). Mice were administered TLE and Boswellia three times a week for 10 weeks. (**A**) Safranin O staining was performed to evaluate the degree of cartilage damage (scale bar = 100 μm). (**B**) The OARSI grading system was used to analyze the degree of OA symptoms. Values represent the mean ± SD and were obtained using the Kruskal–Wallis test followed by Dunn’s post hoc test. (**C**–**E**) Changes in the expression of MMP3, MMP13, and 8-OHdG in the knee joint identified by immunohistochemistry (IHC) were quantified using ImageJ (*n* = 5). Yellow lines indicate tidemarks. Data are displayed as the mean ± standard deviation (SD), calculated using one-way ANOVA and Dunnett’s multiple comparisons test. Statistically significant deviations from the PBS (control) group are indicated as follows: * *p* < 0.05, *** *p* < 0.001, **** *p* < 0.0001. TLE: *Tisochrysis lutea* extract; IHC: immunohistochemistry.

To verify the in vivo function of TLE, we generated DMM-induced OA models and administered TLE orally to mice with OA induced by DMM. The results of Safranin O staining and OARSI grading (Figure 2A,B) show that the protective effect of TLE against OA was higher than that of the positive control Boswellia, a widely used remedy for arthritis in traditional folk medicine [35,36]. Cartilage degradation caused by MMPs may contribute to inflammation, leading to the increased production of ROS [5,37]. Subsequently, we examined whether TLE exhibits anti-catabolic and antioxidant properties in the progression of OA. The immunohistochemistry (IHC) analysis revealed that TLE inhibited the production of MMP3 and MMP13 in DMM-induced OA models (Figure 2C,D). 8-OHdG is specifically generated when the DNA base guanine is oxidized by ROS [38]. IHC experiments conducted on mouse cartilage revealed that TLE reduced 8-OHdG levels (Figure 2C,E), suggesting that TLE not only prevents MMP-induced cartilage destruction but also reduces oxidative stress levels in the cartilage of OA-induced mice.

### 3.3. Fucoxanthin Accounts for 41.2% of TLE

**Figure 3:**
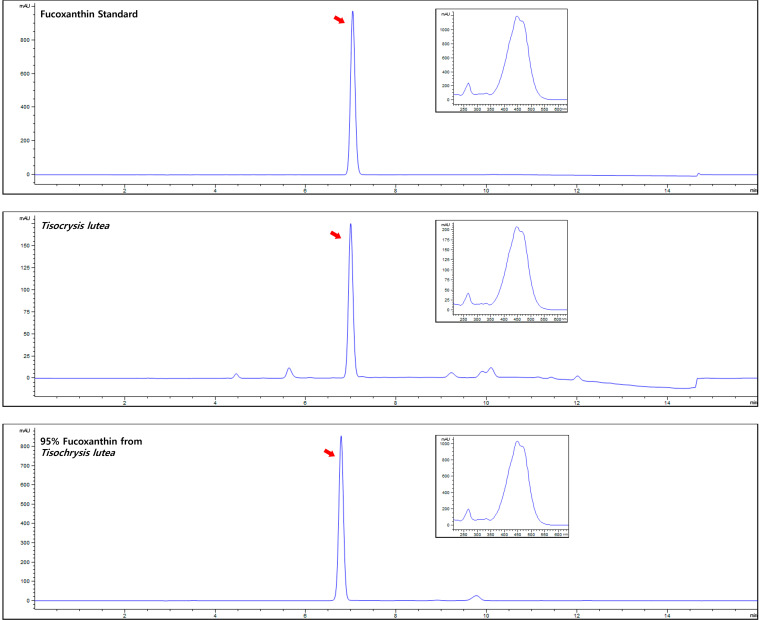
Analysis of fucoxanthin from TLE. High-performance liquid chromatography (HPLC) chromatogram of fucoxanthin standard (**upper**), TLE (**middle**), and fucoxanthin isolated from TLE (**lower**). The elution of samples in each HPLC was analyzed by measuring absorbance at 450 nm, and red arrows indicate the fucoxanthin peak in each chromatogram. The inset displays the absorption spectrum for the peak of fucoxanthin. TLE: *Tisochrysis lutea* extract.

HPLC was used to identify the natural compounds in TLE. A comparative analysis of TLE using HPLC with standard compounds showed that fucoxanthin was the most abundant compound in TLE (Figure 3), with a content of 41.2% (Figure 3, middle panel). To obtain high-purity fucoxanthin, TLE was subjected to silica gel chromatography, and its purity was determined using HPLC (Figure 3, lower). 

### 3.4. Fucoxanthin Inhibits Catabolic Factor Expression

**Figure 4:**
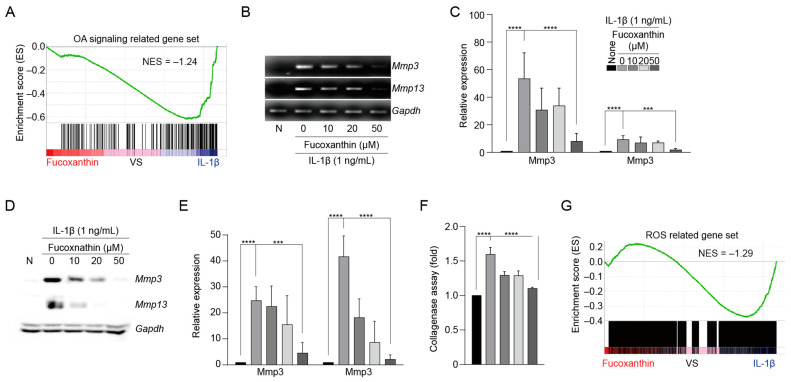
Fucoxanthin isolated from TLE suppresses OA-induced catabolic factor expression. (**A**) GSEA was conducted using RNA-seq data obtained from chondrocytes. (**B**,**C**) Relative mRNA expression levels of MMP3 and MMP13 were determined via PCR and quantified by qRT-PCR (*n* = 5). (**D**,**E**) Protein levels of MMP3 and MMP13 were assessed using Western blot, and their relative intensities were quantified through densitometry. MMP3 molecular weight (MW): 54 kDa. MMP13 MW: 54 kDa. GAPDH MW: 42 and 44 kDa. (**F**) Collagenase activity of MMPs was measured using a collagenase assay. (**G**) GSEA was performed with the ROS gene signature. Data are presented as the mean ± standard deviation (SD), analyzed by one-way ANOVA with Dunnett’s multiple comparisons test (*n* = 5). *** *p* < 0.001, **** *p* < 0.0001. TLE: *Tisochrysis lutea* extract; GSEA: gene signature enrichment analysis.

Chondrocytes were simultaneously treated with IL-1β and fucoxanthin in varying doses, and the expression of catabolic factors was examined through in silico and biochemical methods. First, we performed GSEA to determine whether fucoxanthin inhibited catabolic factor expression under IL-1β-treated in vitro conditions. The gene set in cells under fucoxanthin treatment was positively correlated with a previously reported OA gene signature (Figure 4A). OA-related genes, including those encoding MMP3 and MMP13, are the main factors in cartilage destruction [8,15,16,18]. Here, we found that the transcript levels of MMP3 and MMP13 were suppressed in IL-1β-induced OA mimic conditions (Figure 4B,C). Additionally, we examined whether their protein levels were decreased in fucoxanthin-treated cells. We found that fucoxanthin inhibited MMP3 and MMP13 protein expression and collagenase activity in a dose-dependent manner in IL-1β-treated chondrocytes (Figure 4D–F). GSEA demonstrated that fucoxanthin effectively suppressed ROS production-related gene signature (Figure 4G). Furthermore, CellROX demonstrated that TLE or fucoxanthin reduced ROS production by confocal microscopy analysis in IL-1β-treated chondrocytes (Figure S1), consistent with the GSEA data. Therefore, it can be concluded that TLE-derived 95% pure fucoxanthin has anti-inflammatory activity, suggesting that fucoxanthin has potential as a novel antioxidant for OA therapy. 

### 3.5. Fucoxanthin Suppresses OA Pathogenesis with the Regulation of Genes for ERK and NF-κB Signaling 

**Figure 5:**
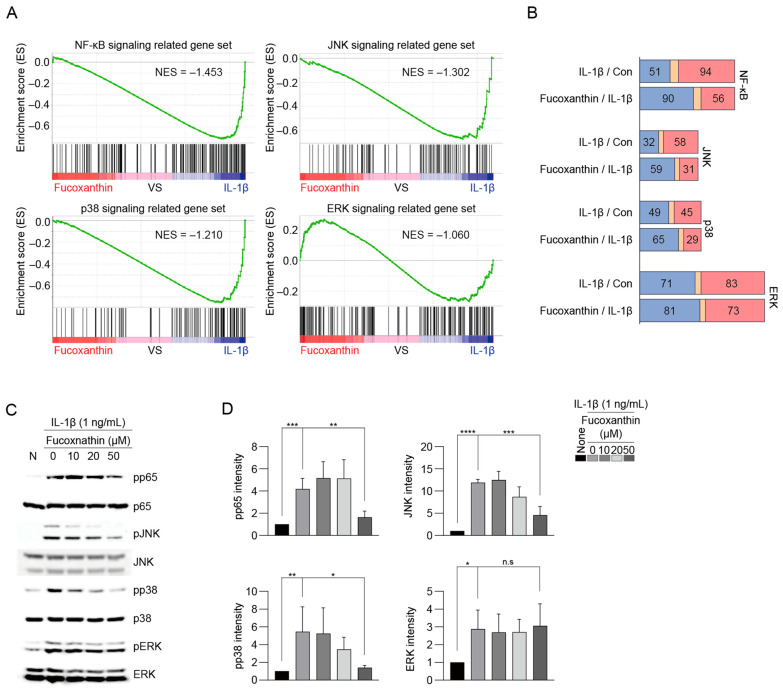
Fucoxanthin isolated from TLE regulates the expression of OA-related signaling molecules through the inactivation of the NF-κB, JNK, and ERK pathways. For the in silico analysis, chondrocytes were administered 50 μM of fucoxanthin sourced from TLE along with IL-1β at 1 ng/mL for 12 h, followed by RNA sequencing. (**A**) RNA-seq was performed using RNA samples isolated from three groups (control, IL-1β, IL-1β + fucoxanthin, 50 μM). GSEA was conducted to analyze the expression of genes associated with each signaling pathway. (**B**) The graph presents the number of genes with an altered expression in samples treated with IL-1β versus that in the control and that in the group with fucoxanthin and IL-1β. (**C**,**D**) Protein levels of ERK, pERK, JNK, pJNK, p65, pp65, p38, and pp38 were detected by Western blotting and their relative intensities were measured using densitometry (*n* = 5). pp65 MW: 65 kDa. p65 MW: 65 kDa. pJNK MW: 46 and 54 kDa. JNK MW: 46 and 54 kDa. pp38 MW: 43 kDa. p38 MW: 40 kDa. pERK MW: 42 and 44 kDa. ERK MW: 42 and 44 kDa. Data are presented as the mean ± SD as determined by one-way ANOVA with Bonferroni’s post hoc test (*n* = 5). Significant differences compared to the IL-1β treated group are denoted as * *p* < 0.05, ** *p* < 0.01, *** *p* < 0.001, **** *p* < 0.0001, and n.s. = not significant. TLE: *Tisochrysis lutea* extract; GSEA: gene signature enrichment analysis).

To uncover the mechanisms behind fucoxanthin’s anti-catabolic effects, RNA sequencing was employed to investigate how it influences genes related to signaling pathways. The NF-κB and MAPK (ERK, JNK, p38) pathways are closely linked to IL-1β signaling. Through in silico analysis, we assessed fucoxanthin’s potential to mitigate osteoarthritis via these routes. We analyzed changes in gene expression associated with these pathways after treatment with fucoxanthin and generated a list of involved genes using IPA. These changes were depicted in a heatmap (Figure 5A,B), focusing on genes whose expression increased in response to IL-1β and comparing their expression levels under fucoxanthin treatment. We counted the genes that showed further upregulation when co-treated with fucoxanthin (Figure 5A,B). Fucoxanthin reduced the expression of 57 out of 72 genes in the NF-κB pathway and 59 out of 81 genes in the ERK pathway (Figure 5B), indicating that fucoxanthin suppressed NF-κB and ERK pathways but not others. Additionally, we correlated these findings with protein expression levels, as visualized in the heatmap, noting that fucoxanthin also reduced NF-κB and pERK protein levels (Figure 5C,D).

**Figure 6:**
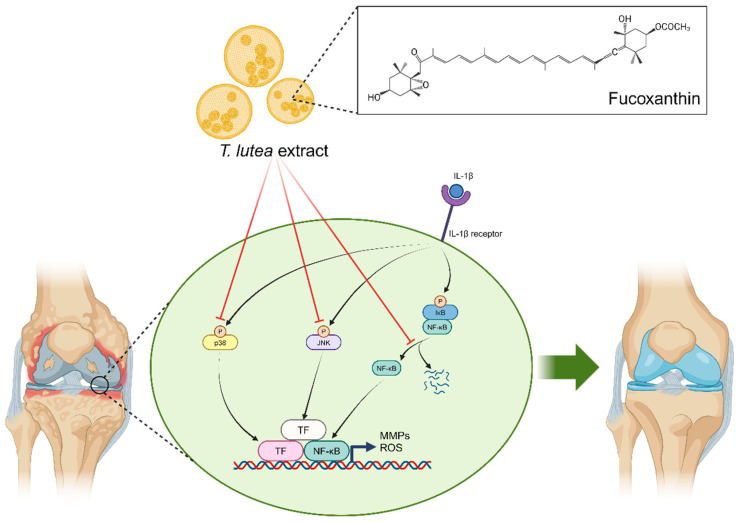
Graphical summary of the action of TLE and fucoxanthin in alleviating catabolic factor expression through the inhibition of NF-κB, JNK, and p38 signaling.

Overall, these results indicate that fucoxanthin comprises 41.2% of TLE and has antioxidant and anti-catabolic effects, which are mediated by the downregulation of the NF-κB, JNK, and p38 signaling pathways (Figure 6).

## 4. Discussion

OA is a persistent and progressive condition affecting the joints, presenting with articular cartilage deterioration and resulting in discomfort, swelling, and diminished joint mobility. Medical treatments mainly focus on alleviating symptoms and include the use of pharmaceuticals like nonsteroidal anti-inflammatory drugs (NSAIDs) and corticosteroids, along with physical therapy; surgical intervention is necessary for severe cases. However, these therapies fail to target the underlying pathology that causes cartilage destruction and frequently result in adverse reactions. Consequently, demand has increased for natural and environmentally friendly alternatives, such as marine microalgae, that provide a plentiful supply of bioactive chemicals with potential therapeutic advantages for OA. Although TLE has anti-inflammatory effects, it is difficult to use as a medical ingredient, as this requires the identification of a specific chemical [39]. We propose the use of TLE as an ingredient in functional foods to reduce cartilage destruction. Therefore, we aimed to assess the viability of TLE as a potential natural therapeutic for OA.

An emerging trend in OA management employs phytotherapy to slow disease progression. This is primarily because phytotherapeutic medicines can reduce the expression of catabolic factors involved in OA pathogenesis and other age-related disorders [39,40,41]. Previous studies have shown that MMP expression can cause significant pathological alterations in the ECM and increase ROS production, which contributes to the development and progression of OA [3,8,16,17]. 

Previous studies have demonstrated that fucoxanthin reduces IL-1β by inhibiting NF-κB, JNK, and p38 signaling pathways [27,42,43,44]. We observed similar effects of fucoxanthin blocking inflammatory responses by inhibiting the effects of IL-1β related to NF-κB, JNK, and p38 signaling pathways. We confirmed that fucoxanthin from TLE inhibited the phosphorylation of JNK and p38 and, subsequently, NF-κB signaling in IL-1β-treated chondrocytes. Fucoxanthin is a specific carotenoid present in brown algae, particularly in marine sources [26]. Fucoxanthin is an anti-catabolic substance similar to vitamins C and E, carotenoids, and flavonoids. However, its distinct sources, specific health benefits, and mechanisms of action set it apart from the broader category of functional dietary ingredients [45]. 

Based on the HPLC results, the oral administration of TLE at a concentration of 100 mg/kg in our study is equivalent to approximately 41.2 mg/kg of fucoxanthin. Additionally, considering the aforementioned studies, it is likely that the serum levels of fucoxanthinol were increased in the mice that ingested TLE [46,47]. Additionally, fucoxanthinol, the primary metabolite of fucoxanthin, has a half-life of approximately 7 h in human plasma following oral administration. This was observed in studies by Hashimoto et al. [48] and corroborated by Mok et al. [49]. The maximum concentration typically reaches around 4 h post-administration, after which the concentration decreases gradually. These previous studies are expected to aid in the pharmacokinetic understanding of fucoxanthin derived from TLE. However, although we proved the effect of fucoxanthin in IL-1β-treated chondrocytes, we were not able to demonstrate the in vivo effect of fucoxanthin in DMM mice. These data cannot clearly support the theory that the in vivo effect of TLE is due to fucoxanthin. We should conduct additional in vivo research on fucoxanthin to validate this issue in the near future.

This study aimed to examine the effects of TLE as a natural therapeutic agent for preventing OA. *T. lutea* is a marine microalga that contains a wide range of bioactive chemicals, making it an interesting prospect for phytotherapy research. We investigated the efficacy of TLE as a natural fucoxanthin source. Additionally, we found that TLE and its predominant ingredient, fucoxanthin, are organic compounds that can prevent OA by inhibiting processes that break down the cartilage. Our investigation revealed that the oral administration of TLE reduced OA progression by reducing cartilage damage. Furthermore, we discovered that the TLE-derived compound fucoxanthin inhibited the MAPK and NF-κB signaling pathways, thereby reducing the production of catabolic factors (MMP3, MMP13, and ROS). Therefore, we suggest that the incorporation of TLE or fucoxanthin into therapeutic agents could be a potential avenue to treat OA. However, further investigation is needed to determine whether fucoxanthin produces this effect by directly blocking the IL-1 receptor, which is similar to an IL-1 receptor antagonist. According to previous studies, synoviocytes are also affected by IL-1β, leading to similar effects on the cartilage in OA as chondrocytes [49]. Therefore, *T. lutea* and fucoxanthin may show similar effects on synoviocytes by inhibiting IL-1β-induced inflammatory responses. Moreover, previous reports demonstrated that the oral administration of 65 mg/kg of fucoxanthin in rats for 30 min resulted in the rapid conversion of fucoxanthin to fucoxanthinol in the plasma [50,51]. Additional research is needed to confirm the effectiveness of fucoxanthin in treating patients with OA. We anticipate that the findings of this study, along with future research, will show that TLE and fucoxanthin can effectively address the underlying causes of OA in humans. 

## 5. Conclusions

In this study, we found that the extract of TLE contained mainly fucoxanthin. In vitro and in vivo studies demonstrated that TLE protects against cartilage destruction by inhibiting catabolic activity. Furthermore, fucoxanthin inhibited upregulated OA-associated genes through regulating the MAPK and NF-κB signaling pathways. Collectively, these results highlight the potential of TLE as a source of fucoxanthin, an antioxidant, for use as a therapeutic agent for OA treatment.

## Data Availability

The data supporting the findings of this study are provided within the article and its Supplementary Materials.

## Supplementary Materials

The following supporting information can be downloaded at: https://www.mdpi.com/article/10.3390/antiox13080941/s1, Figure S1. ROS in chondrocytes was detected using the CellROX green reagent.
