# Supplementary material for: Tisochrysis lutea Fucoxanthin Suppresses NF-κB, JNK, and p38-Associated MMP Expression in Arthritis Pathogenesis via Antioxidant Activity
Source: Antioxidants (Basel). 2024 Aug 2;13(8):941. doi: 10.3390/antiox13080941 (PMC11351224; doi:10.3390/antiox13080941)
Supplement: Supplementary file 1 [file antioxidants-13-00941-s001.zip › antioxidants-3077129-supplementary.pdf]

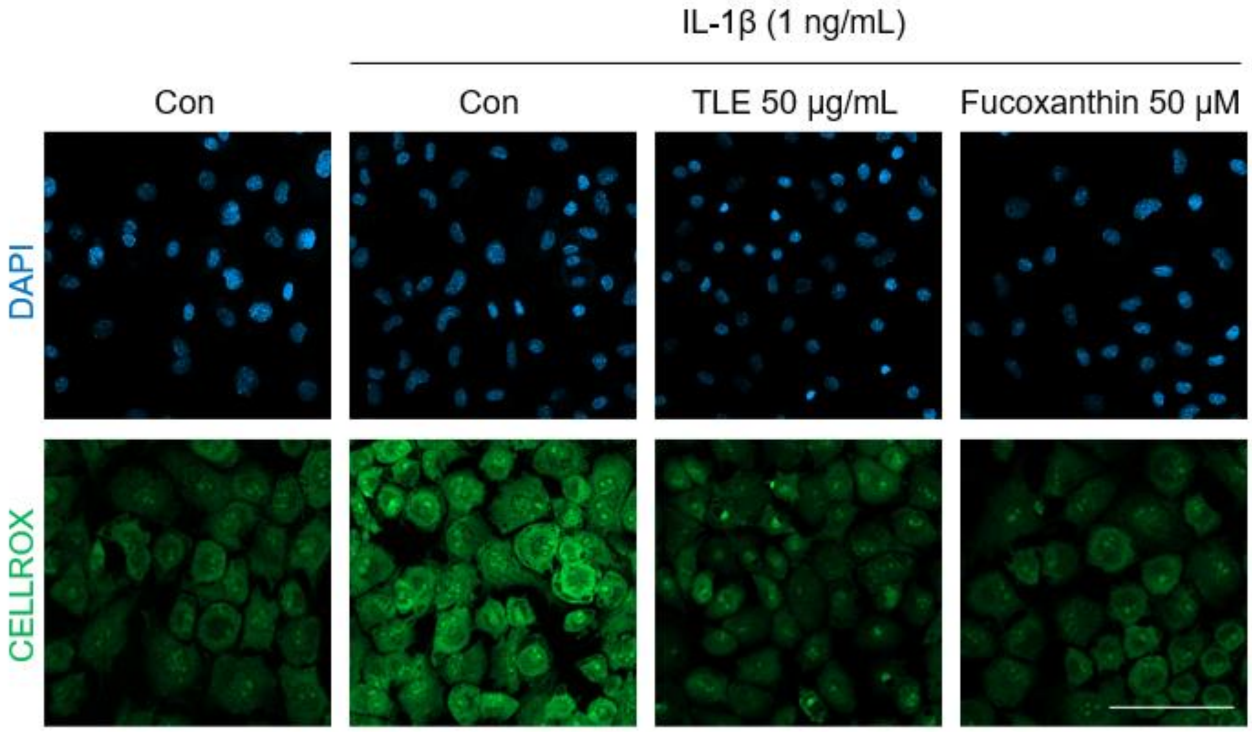

Figure S1. ROS in chondrocytes was detected using the CellROX green reagent. After treating IL-1β-treated cells with TLE and fucoxanthin, respectively, the cells were stained with CellROX and DAPI. Images were captured using confocal microscopy. Scale bar = 100 μm.
